# Whole-Body MRI versus bone scintigraphy: which is the best diagnostic tool in patients with chronic recurrent multifocal osteomyelitis (CRMO)?

**DOI:** 10.1186/1546-0096-13-S1-P58

**Published:** 2015-09-28

**Authors:** MF Villani, L Tanturri de Horatio, MC Garganese, I Casazza, S Savelli, M Pardeo, V Messia, F De Benedetti, A Insalaco

**Affiliations:** 1 Bambino Gesù Children Hospital, Nuclear Medicine Unit, Rome, Italy; 2 Bambino Gesù Children Hospital, Department of Radiology, Rome, Italy; 3 Bambino Gesù Children Hospital, Pediatric Medicine-Rheumatology, Roma, Italy

## Introduction

Chronic recurrent multifocal osteomyelitis (CRMO) is an autoinflammatory bone disorder of unknown etiology with a wide range of clinical manifestations. Since CRMO is a systemic disorder that can affect multiple skeletal sites, whole-body imaging techniques (whole body bone scintigraphy -WBBS- or whole body magnetic risonance -WBMRI-) provide major contribution to the initial diagnostic approach, as well as during follow-up.

## Objective

To compare WBMRI with WBBS in the assessment of bone lesions in patients with CRMO.

## Materials and methods

We retrospectively evaluated all WBMRI examinations performed between January 2010 and December 2014 in 18 patients with clinical, laboratory and histology findings suggestive for CRMO.WBMRI were evaluated independently by two experienced paediatric radiologists, who eventually reached consensus. All patients also underwent WBBS within four weeks; WBMRI and WBBS findings were compared. Signal hyperintensity compared with normal bone on STIR images and increased tracer activity in bone delayed scans on WBBS were considered indicative of disease involvement.

## Results

WBMRI and WBBS showed 225 and 132 lesions, respectively. In the appendicular skeleton, WBMRI demonstrated 143 lesions and WBBS 66 lesions; in the axial skeleton, WBMRI demonstrated 63 lesions and WBBS 18 lesions. WBMRI demonstrated joint involvement in 19 sites and WBBS in 48. The higher concordance between the two methods was observed in the sacroiliac joint (13 lesions for both methods); the higher discordance was observed in spine (WBMRI showed 55 lesions and WBBS 5) and in sterno-clavicular joint (WBMRI showed 2 lesions while WBBS 12).

## Conclusion

Both WBBS and WBMRI confirmed to be useful tools for the detection of CRMO lesions, as it is reported in literature. Discordance between WBBS and WBMRI is probably due to several factors. In the evaluation of axial skeleton (in particular of the spine), WBMRI shows higher spatial resolution than planar WBBS, but tomographic acquisition (SPECT) enhances the sensitivity of WBBS. Moreover, age-related conversion of hematopoietic marrow to fatty marrow in children may create a confusing appearance on MRI and may be misleading. Finally, in particular in WBMRI performed during treatment and follow-up, clinical relevance of the WBMRI-positive but WBBS-negative lesions is unclear, as still debated in current literature.

